# Mechanism of Water Intrusion into Flexible ZIF-8: Liquid Is Not Vapor

**DOI:** 10.1021/acs.nanolett.3c00235

**Published:** 2023-06-09

**Authors:** Eder Amayuelas, Marco Tortora, Luis Bartolomé, Josh David Littlefair, Gonçalo Paulo, Andrea Le Donne, Benjamin Trump, Andrey Andreevich Yakovenko, Mirosław Chorążewski, Alberto Giacomello, Paweł Zajdel, Simone Meloni, Yaroslav Grosu

**Affiliations:** † Centre for Cooperative Research on Alternative Energies (CIC energiGUNE), Basque Research and Technology Alliance (BRTA), Alava Technology Park, Albert Einstein 48, 01510 Vitoria-Gasteiz, Spain; ‡ Dipartimento di Ingegneria Meccanica e Aerospaziale, Sapienza Università di Roma, via Eudossiana 18, 00184 Rome, Italy; § Dipartimento di Scienze Chimiche e Farmaceutiche (DipSCF), Università degli Studi di Ferrara (Unife), Via Luigi Borsari 46, I-44121 Ferrara, Italy; ∥ NIST Center for Neutron Research, National Institute of Standards and Technology, Gaithersburg, Maryland 20899, USA; ⊥ X-Ray Science Division, Advanced Photon Source, Argonne National Laboratory, Argonne, Illinois 60439, USA; ∇ Institute of Chemistry, University of Silesia, Szkolna 9, 40-006 Katowice, Poland; ¶ Institute of Physics, University of Silesia, 75 Pulku Piechoty 1, 41-500 Chorzow, Poland

**Keywords:** synchrotron radiation, MOFs, intrusion mechanism, hydrophobic surfaces, molecular dynamics

## Abstract

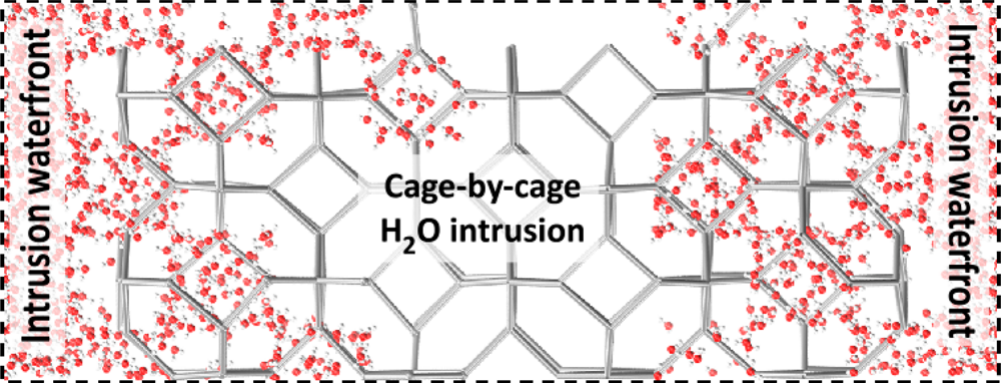

Zeolitic Imidazolate Frameworks (ZIF) find application in storage and dissipation of mechanical energy. Their distinctive properties linked to their (sub)nanometer size and hydrophobicity allow for water intrusion only under high hydrostatic pressure. Here we focus on the popular ZIF-8 material investigating the intrusion mechanism in its nanoscale cages, which is the key to its rational exploitation in target applications. In this work, we used a joint experimental/theoretical approach combining in operando synchrotron experiments during high-pressure intrusion experiments, molecular dynamics simulations, and stochastic models to reveal that water intrusion into ZIF-8 occurs by a cascade filling of connected cages rather than a condensation process as previously assumed. The reported results allowed us to establish structure/function relations in this prototypical microporous material, representing an important step to devise design rules to synthesize porous media.

Wetting and dewetting of microporous media, i.e., materials with pores diameter smaller than 2 nm, is of great importance for broad range of technological and natural systems.^1,2^ Among others, energy-absorbing and energy-storing systems have attracted considerable attention in recent years as they are expected to play a crucial role in a future carbon free society.^3^ Many of these systems transfer mechanical energy through pressurized intrusion–extrusion cycles of a liquid into/out of a porous material.^4,5^ The wetting and dewetting of a microporous material is a truly multiscale process, in which the emerging characteristics of the intrusion/extrusion cycle exhibit a nontrivial dependence on physio-chemical details at the molecular level: the type of material, its structure and morphology and its chemical composition.^6,7^ Thus, a microscopic understanding of the intrusion and extrusion processes is key to design novel, high-performance materials.

In this context, metal–organic frameworks (MOFs) have arisen as a promising class of materials to achieve breakthrough performance in energy absorption during mechanical impact.^8−11^ The large inner surface area (up to 10,000 m^2^g^–1^), the highly tunable framework architecture, and the chemical composition of MOFs^12^ make these materials attractive candidates for energy-dissipation systems. Among the vast number of MOFs, only a handful have been studied for this application,^13−16^ owing to their good chemical, thermal, and mechanical stability, and the relatively inexpensive chemicals and synthesis protocol for their production; they predominantly belong to the hydrophobic microporous family of zeolitic imidazolate frameworks (ZIFs).^17^ Arguably, the most extensively studied microporous material to date is ZIF-8, which has become a reference for microporous materials in the area of water intrusion–extrusion and related applications.^8,15,18−20^ Traditionally, research in the field focused on the empirical evaluation of energy absorption performance of ZIF-8 and other microporous media. However, attention has recently turned toward the investigation of the microscopic mechanism of hydrophobic ZIF wetting.^21^ Deepening the understanding of hydrophobic wetting has been the main goal of several groups in the scientific community in recent years, exploring the effects of confining water in hydrophobic nanopores, the interactions of water with different hydrophobic surfaces, and the liquid and vapor interfaces in nanoconfinement under hydrostatic pressure.^22−27^ In a recent work, Sun et al.,^28^ proposed the intrusion of ZIF-8 to be governed by the condensation of vapor present in the nanoscale cages, postulating that the kinetics of the intrusion process is determined by the intrinsic length (single nanometer) and time scales (nanoseconds) necessary for critical water clusters to nucleate inside individual cages.

Understanding the key rate-limiting step of intrusion is of paramount importance to design improved materials for energy dissipation and the key contribution of Sun et al.^28^ paved the way to such investigation. Indeed, the hysteresis, i.e., the difference of thermodynamic conditions between intrusion and extrusion of a nanoporous material, determines how much energy per intrusion-extrusion cycle is dissipated. Hysteresis, in turn, depends on how quickly the system reaches the equilibrium state corresponding to the given thermodynamic conditions, whose microscopic determinants are investigated here.

In this work, combining intrusion experiments, in operando synchrotron radiation, atomistic simulations, classical continuum theories, and stochastic models, we report some important insights into the multiscale mechanism of ZIF-8 MOF wetting by H_2_O. We reveal that the intrusion of water in ZIF-8 proceeds by the cascade penetration of water in connected cages, forming coherent domains of ZIF-8 cages with alike structural characteristics. This mechanism is fundamentally different from the previously proposed condensation-driven intrusion in individual ZIF-8 cages. We show that the proposed cascade intrusion mechanism crucially depends on the hydrogen bonds which form across neighboring cages, which would instead be irrelevant in the condensation scenario. On a larger scale, water intrusion progresses by the formation and growth of coherent domains of wet cages, which progressively and cooperatively advance through the MOF crystallite. Our conclusions support new design criteria for MOFs for energy applications, hinging on pore connectivity rather than on individual cage properties.

First, we focus on structural changes in ZIF-8 (flexible sodalite, SOD, topology; Figure 1c)^29^ during water intrusion at 30 °C by in operando synchrotron radiation and intrusion porosimetry. Figure 1a shows the evolution of the unit cell parameter *a* during the experiment, namely the expansion of *a* during H_2_O intrusion (Figure S1). We have already reported the negative compressibility of ZIF-8 upon intrusion in a recent article.^21^ The analysis of the shape of the diffraction pattern peaks allowed us to identify the strain (Figure 1b) produced in ZIF-8 (Basolite Z1200) under hydrostatic pressure scans (further experimental details in the Supporting Information). Additionally, we were able to identify the coherent crystalline domain size, i.e., the largest domain of crystallites with the same structural characteristics. In fact, wet cavities are larger than dry ones (Figure 1d), and if, during intrusion, domains of fully wet and completely dry cages are formed (Figure 1e), these can be detected by synchrotron radiation and the size of the largest one determined.

**Figure 1:**
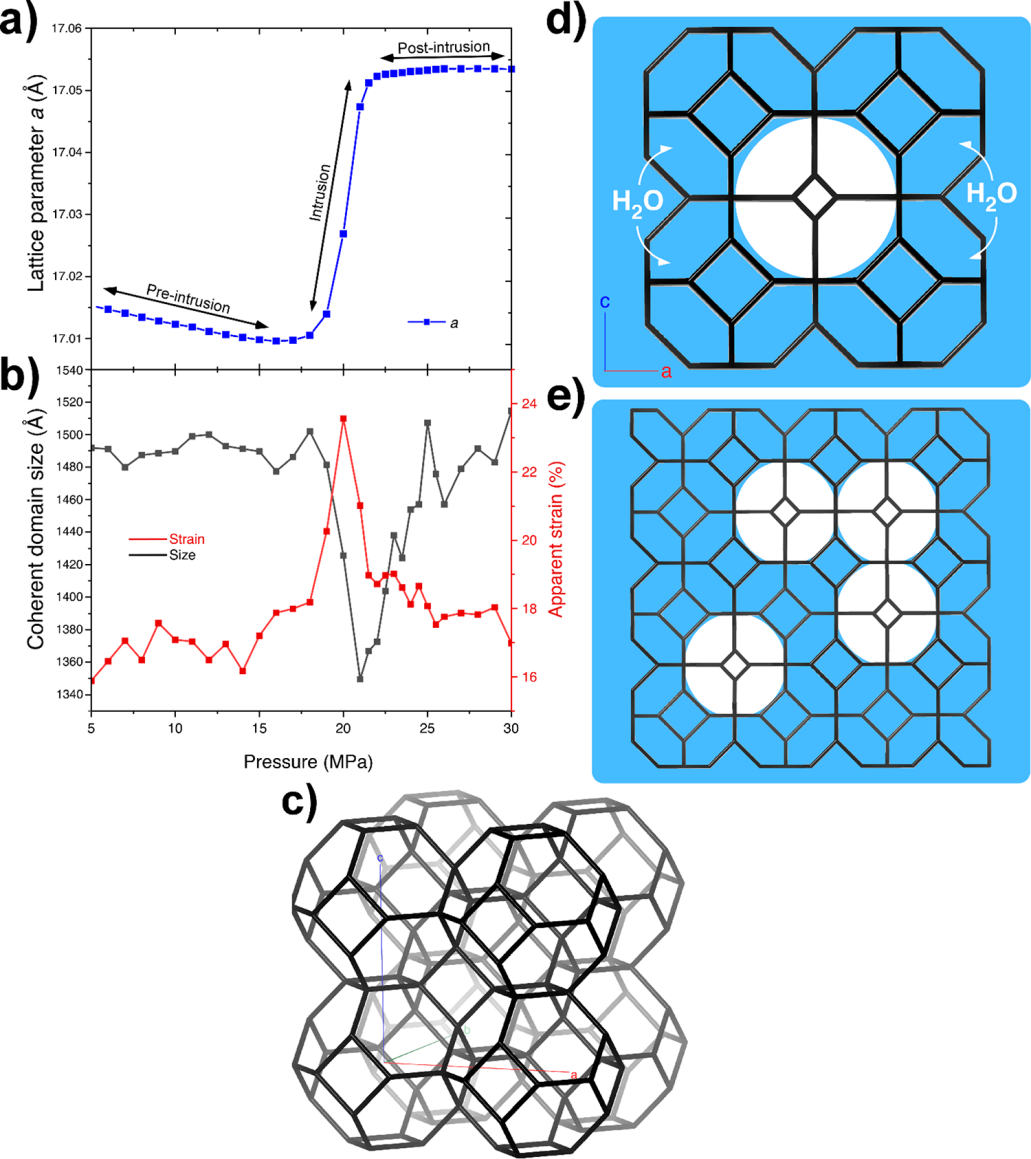
a) Evolution of lattice parameter during H_2_O intrusion at 30 °C. b) Evolution of apparent strain and coherent crystalline domain size versus pressure for ZIF-8 + water system at 30 °C. c) SOD topology of ZIF-8 showing the interconnected cavities. d) Schematic simplification of the wetting of external cavities in a ZIF-8 crystal, becoming larger than the dry ones (blue color stands for water. e) Wetting process of an extended ZIF-8 structure, with different domain sizes: the wet ones larger (blue) and the dry ones smaller (white). We remark that the cartoon shown in this panel is purely illustrative: synchrotrons do not allow us to identify the geometry of wet/dry domains, whether cubic-like, spherical-like or more complex, nor if a single or multiple wet/dry domains are formed during intrusion.

Both the strain and the size of coherent domains present a sharp variation in correspondence of the intrusion process (compare Figure 1a,b, and Figure S1). Considering that wet ZIF-8 cages undergo an expansion relative to dry ones,^21^ intrusion introduces some strain in partly filled ZIF-8 crystallites due to the size mismatch between wet and dry cages (Figure 1e). In other words, expansion of wet cages during intrusion breaks the local symmetry introducing a strain. This strain continuously increases with the number of wet cages up to a maximum. We hypothesize that this maximum is attained when the number of wet cages equals that of dry ones: in this condition one should have the maximum mismatch and strain.

Simultaneously to the increase of strain one observes a decrease in the size of the largest coherent domains. Our interpretation of this empirical evidence is that wet cages form continuous, coherent domains—in a broad sense “droplets”—within the initially empty ZIF-8 crystallites. These droplets grow up to the point that the coherent domain of dry cages (“bubbles”) becomes the smallest coherent domain in the ZIF-8 crystallite; from this point on, the largest coherent domain becomes the wet one, which steadily grows (Figure 1b) while the dry domain shrinks.

Summarizing, synchrotron data suggest that ZIF-8 is intruded by water following a cascade mechanism by progressive filling of connected cages, which account for the observed shrinkage and growth of coherent domains.

To clarify the molecular mechanism of wetting of complex microporous systems, we performed molecular dynamics atomistic calculations in which a *z*-oriented ZIF-8 slab is immersed in bulk water, Figure 2a; a 25 MPa pressure is applied by two pistons parallel to the slab, according to the method proposed by Marchio et al.^30^ Time scales accessible by atomistic simulations, tens of nanoseconds, are too short to observe the wetting of the slab; thus, the process was accelerated using the restrained molecular dynamics (RMD). RMD forces the system to visit the microscopic states corresponding to an overall level of wetting of the ZIF-8 computational sample as measured by the number of water molecules within the MOF slab. It is worth remarking that these microscopic states are sampled with a statistical distribution consistent with the constant number of particles, pressure, and temperature ensemble and in quasi-static conditions, i.e., in conditions consistent with the experimental ones (see details of the method and the computational setup in the Supporting Information). RMD also allows us to compute the free energy profiles characterizing the wetting process.^21^ At a variance with previous simulations focusing on a three-periodic ZIF-8 bulk sample, with water condensation investigated by Grand Canonical Monte Carlo simulations,^28^ in which new water atoms are inserted at random locations within the material, our approach allows ZIF-8 wetting to take place according to most probable mechanism without excluding a priori neither condensation nor cascade penetration.

**Figure 2:**
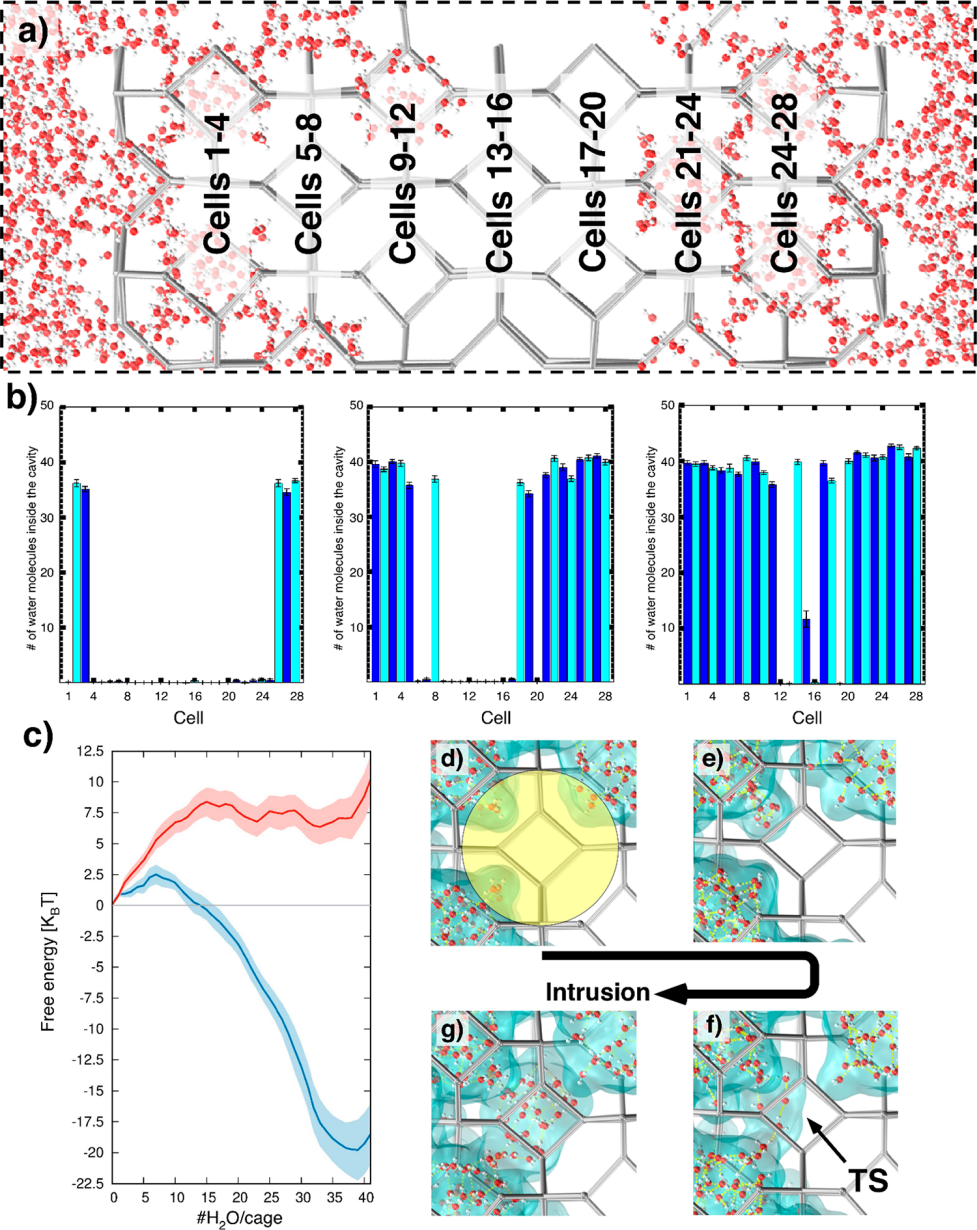
a) Computational ZIF-8 sample, made by a (100) oriented slab comprising 7 layers of ZIF-8 cages. Each layer contains 4 cages, for a total of 28 complete cages. Cages are numbered in ascending order starting from the left layer of the slab, as indicated in the sketch. b) Number of water molecules per cage during the intrusion process at three levels of overall filling. These histograms show that intrusion starts from cages in contact with bulk water (cages 1–4 and 25–28) and proceeds toward the interior through a cascade, cage-by-cage filling mechanism. c) Free energy profile of wetting for the same ZIF-8 cage (denoted by the yellow circle in panel d) when (i) water is allowed to enter by all 5 wet cages surrounding the wetted one (blue) or (ii) water is allowed to enter only from one of them (red). The shadowed regions denote the (statistical) error, standard deviation. The difference between the two curves, well beyond the error, confirms that wetting from neighboring cages is favored as compared to water condensation, the latter depending only on the number of water molecules in the cage. d–g) Snapshots of the single cage wetting process in blue. Panel f corresponds to the transition state (TS) of the single cage intrusion process, the state corresponding to the maximum of the free energy (see also Figure S2 in the Supporting Information).

To identify the actual wetting mechanism, we computed the number of molecules in each of the 28 cages comprising the ZIF-8 slab (Figure 2a). If intrusion took place via condensation in bulk cages, one should observe water molecules in the gas phase permeating the slab and condensing at random cages within the material. On the contrary, one notices that the wetting of the slab starts by the complete filling of specific cages belonging to the outermost layers, those in contact with bulk water, and proceeds by filling cages connected to the wet ones through 6MR apertures, which is consistent with the cascade intrusion mechanism proposed above.

To further clarify the ZIF-8 wetting mechanism, we simulated water intrusion in a single cage. We started from the partly filled ZIF-8 slab in the state containing ∼960 water molecules and ran additional RMD simulations to refine the free energy profile for the filling of an individual cage (blue line in Figure 2c). Before the intrusion of this cage starts, i.e., when the number of water molecules #H_2_O/cage is zero, some water molecules inside the surrounding wet cages are present near the connecting 6MR apertures. When wetting starts, i.e., when #H_2_O/cage grows, these molecules penetrate the cage and the free energy grows. This can be explained considering that in this early stage the penetrating water molecules are undercoordinated; i.e., they form fewer hydrogen bonds than in the initial state.^31^ This energetically unfavorable process is driven by the external pressure. The free energy ceases to increase at the transition state (free energy maximum) which occurs when water molecules penetrating from neighboring wet cages can form hydrogen bonds between them (Figure 2f), which suddenly reduce the energy penalty associated with H_2_O undercoordination. This event requires that enough water molecules penetrate the cage, ca. 8 water molecules according to our simulations. From this point onward, the free energy decreases until a minimum is reached at #H_2_O/cage ∼38, which is consistent with the typical water molecule occupancy obtained from the histograms of Figure 2b for fully filled cages.

To confirm that the wetting mechanism is due to cascade penetration rather than condensation, we performed additional free energy calculations for the wetting of a single cage but allowing water to enter from only one of the surrounding wetting cages (red line in Figure 2c). If wetting is by condensation, with the number of water molecules in the liquid-like water nucleus as the “reaction coordinate”, there should be no significant difference between the free energy profiles in the two cases. Instead, Figure 2c shows that allowing water to enter in a cage from one or multiple surrounding cages completely changes the free energy profile. Specifically, it is energetically more convenient to form a water bridge across several 6MR apertures with already filled ZIF-8 cavities. This finding confirms our hypothesis that the kinetically favored mechanism to wet ZIF-8 is through cascade penetration, with water penetrating from cage to cage, with the transition state corresponding to the formation of hydrogen bonds among water molecules entering from neighboring cages (Figure 2f, see also Figure S2). This brings us to propose that the key structural parameter controlling hysteresis is the pore connectivity, including the number and distance of connecting apertures through which water molecules can propagate capillary penetration. To some extent, these key characteristics are related to the cage size of the MOF, but the latter characteristic is not as important for determining hysteresis via limiting the size of critical liquid-like water nuclei, as previously proposed.^28^

The results of the single cage wetting simulation clearly show that the microscopic ZIF-8 wetting mechanism is not condensation but that the transition state is determined by the formation of a hydrogen bond bridge between water molecules penetrating from neighboring wet cages, rather than by the competition of free energy gain corresponding to larger liquid-like water clusters and the free energy penalty, which characterize condensation processes (see Supporting Information for a summary of classical models of condensation processes).

The simulated microscopic penetration mechanism suggests that there is a correlation between the wetting state of neighboring cages, which can favor the formation of coherent domains of wet or dry cages, causing in turn different structural and mechanical characteristics (strain). It is helpful to explain the formation of these coherent domains in terms of the macroscopic theory of capillarity. Let us consider the case of *n* fully wet cages (∼40 H_2_O per cage) and focus on two cases, (i) one in which the wet cages are randomly distributed in the ZIF-8 crystallite and (ii) the other in which they form a single, droplet-like, coherent domain. According to our recent finding that water molecules can form hydrogen bonds across secondary apertures of porous systems,^32^ the droplet-like state is energetically favored. MD simulations reveal that hydrogen bonding across 6MR apertures stabilizes the system by 6.5 *k*_B_*T* (see the Supporting Information) per pair of adjacent wet cages connected by the aperture. Thus, the droplet-like configuration is energetically favored because it minimizes the number of wet cages in contact with dry ones. Indeed, the number of “interface” wet–dry 6MR apertures is proportional to the surface of the coherent domain of wet cages which tends to be minimized for energetic reasons. It is worth remarking that the argument developed here is analogous to the one at the basis of the capillarity, which predicts that isolated droplets are spherical to minimize surface energy; similarly to classical capillarity,^33^ intrusion in ZIF-8 is controlled by a competition between bulk-like volume terms favoring intrusion—mainly the liquid pressure—and the surface cost related to the interface of wet–dry cages which introduces a (free) energy penalty that disfavors the formation of many small wet domains.

Atomistic simulations, with their (relatively) small computational sample preclude a direct empirical confirmation of the wetting mechanism on the crystallite scale. Thus, we developed a stochastic model in which cages undergo wetting or drying with a probability depending on the number of wet neighbors, coherently with the results in Figure 2c, with a ∼2.5 *k*_B_*T* reduction of the wetting barrier per surrounding wet cage (see Supporting Information for the details). The simulated crystallite has the same type of connectivity of ZIF-8. Mimicking experiments, in our model, we linearly increase the pressure in a ramp, although for computational reasons we use compression rate orders of magnitude faster than the typical experimental ones (10^6^ MPa/s vs 3.16 × 10^–6^ MPa/s). The results of our model, shown in Figure 3, confirm the conclusion that the stabilization provided by hydrogen bonding across 6MR apertures results in the formation of one large coherent domain (see Supporting Information for a full movie of the process) while the random filling of cages by condensation is negligible. Because of the high rate at which the pressure is changed, the system forms straight “liquid fronts” connected by curved edges which bear memory of the square geometry and of the initial conditions. In an infinitely long calculation, one would reach the surface with minimal energy—a single spherical empty domain in the center. Additionally, we remark that the advancing flat front of Figure 3, or the single spherical domain mentioned in the previous sentence, is the result of some simplification adopted in the stochastic model, which is based on a defect-free, perfectly cubic, ZIF-8 crystallite, with all wet cages containing the same amount of liquid. This might change the fine details of the mechanism, whether the advancing from is regular or not, but not the overall, cage-by-cage intrusion mechanism.

**Figure 3:**
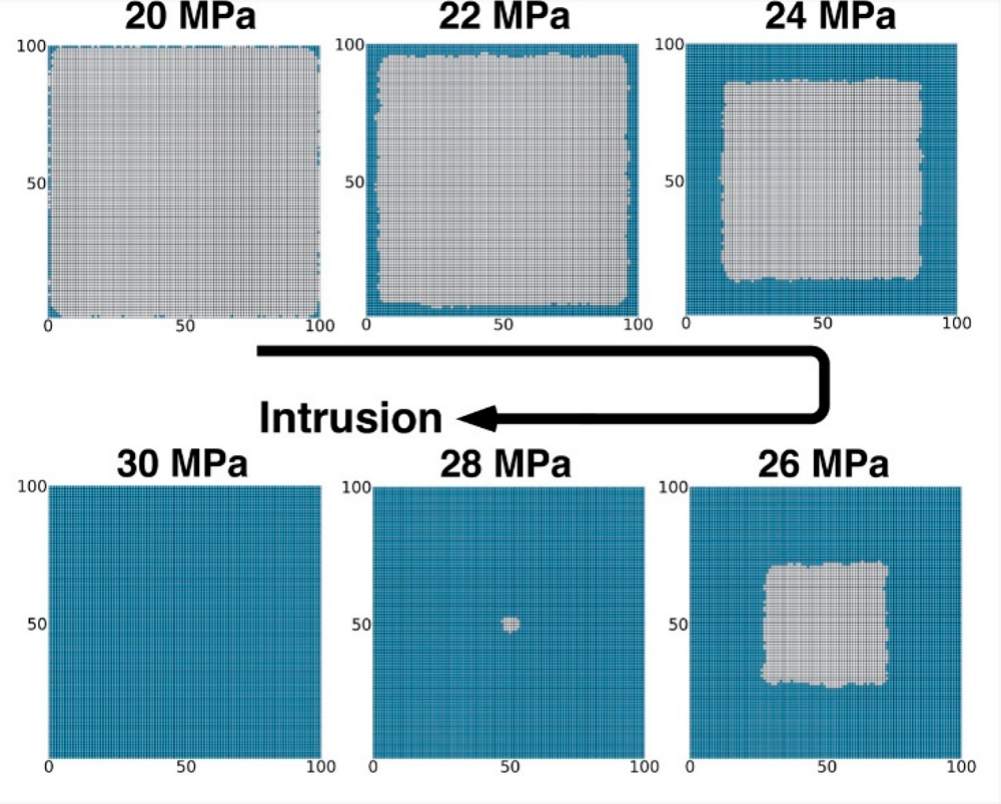
Stochastic model of water intrusion in a ZIF-8 cubic crystallite. Gray and blue squares represent dry and wet cages, respectively. One notices that, as pressure increases, water starts to intrude in the crystallite. Here, intrusion takes place according to a cascade penetration mechanism forming a small number of growing “droplets” of wet cages. These droplets eventually merge, and water completely fills the crystallite.

In summary, via a combination of experimental and theoretical approaches we have clarified the intrusion mechanism of ZIF-8 under hydrostatic pressure, which proceeds by cascade filling of neighboring cages. In operando synchrotron radiation tracked the evolution of domains of wet/dry cages during intrusion, indicating the formation coherent domains of wet cages which grow during the process. RMD simulations gave microscopic evidence of this process, bringing to the conclusion that coherent domains are formed due to penetration of water in connected ZIF-8 cages, rather than water condensation in individual ones, as previously proposed. The detailed wetting mechanism of a single cage has been analyzed, showing that the kinetic bottleneck of the process is the formation of hydrogen bonds bridging water molecules across neighboring apertures. This suggests that tuning the intrusion or extrusion characteristics of hydrophobic MOFs can be obtained by optimizing the pore connectivity; in particular, the geometrical and chemical characteristics of the apertures are crucial to favor or disfavor the formation of hydrogen bonds, which, in turn, drive the cascade filling of neighboring cavities. Finally, on the crystallite scale, the shape of the domains of wet cages seems compatible with the classical capillarity concept of surface energy because they tend to minimize the surface area. Indeed, a stochastic model informed by MD simulations confirms the prevalence of cascade penetration in coherent domains as opposed to a condensation scenario.

The critical characteristics responsible for the water intrusion mechanism if ZIF-8 is the presence of interconnected cages. Thus, we expect a similar mechanism to hold also for other ZIF MOFs. For example, for ZIF-67, which is an isomorph to ZIF-8, simulations show cage-by-cage intrusion (Figure S5). Other ZIFs, e.g., ZIF-12, with different morphologies and cage and aperture sizes, might show some differences with respect to the mechanism discussed above, which, however, we expect to be quantitative rather than qualitative. We plan to investigate this in the future.

## Supporting Information Available

Experimental porosimetry and synchrotron details and data, PV isotherm, atomistic simulations details, density maps, average volume of a ZIF-8 cage along intrusion, schematic model of the mechanism of water condensation, number of water molecules per cage during the intrusion process, and stochastic model additional information (PDF)
