# Supplementary material for: Mechanism of Water Intrusion into Flexible ZIF-8: Liquid Is Not Vapor
Source: Nano Lett. 2023 Jun 9;23(12):5430–6. doi: 10.1021/acs.nanolett.3c00235 (PMC10311589; doi:10.1021/acs.nanolett.3c00235)
Supplement: Supplementary file 1 — nl3c00235_si_001.pdf [file nl3c00235_si_001.pdf]

On the mechanism of water intrusion into flexible ZIF-8: liquid is not vapor

Eder Amayuelas,[a] Marco Tortora,[b] Luis Bartolomé, [a] Josh David Littlefair,[c] Gonçalo Paulo,[b] Andrea Le Donne,[c] Benjamin Trump,[d] Andrey Andreevich Yakovenko,[e] Mirosław Chorążewski,[f] Alberto Giacomello,[b],* Paweł Zajdel,[g],* Simone Meloni,[c],* Yaroslav Grosu.[a],[f],*

a Centre for Cooperative Research on Alternative Energies (CIC energiGUNE), Basque Research and Technology Alliance (BRTA), Alava Technology Park, Albert Einstein 48, 01510 Vitoria-Gasteiz, Spain

b Dipartimento di Ingegneria Meccanica e Aerospaziale, Sapienza Università di Roma, via Eudossiana 18, 00184 Rome, Italy

c Dipartimento di Scienze Chimiche e Farmaceutiche (DipSCF), Università degli Studi di Ferrara (Unife), Via Luigi Borsari 46, I-44121, Ferrara, Italy

d NIST Center for Neutron Research, National Institute of Standards and Technology, Gaithersburg, Maryland 20899, USA

e X-Ray Science Division, Advanced Photon Source, Argonne National Laboratory, Argonne, Illinois 60439, USA

f Institute of Chemistry, University of Silesia, Szkolna 9, 40-006 Katowice, Poland

g Institute of Physics, University of Silesia, 75 Pulku Piechoty 1, 41-500, Chorzow, Poland

Experimental

Materials

All chemicals were used as received from reliable commercial sources. Basolite® Z1200 (ZIF-8 material) was purchased from Sigma-Aldrich Co.

Porosimetry experiments

For water porosimetry commercial ZIF-8 was mixed with water and encapsulated into flexible, hermetic polimeric capsule prior to testing. An Auto Pore IV 9500 porosimeter (Micromeritics Instrument Corporation, Norcross, USA) was used for the compression tests, where the penetrometer was evacuated to a pressure less than 7 Pa, followed by filling with mercury to 50 MPa.

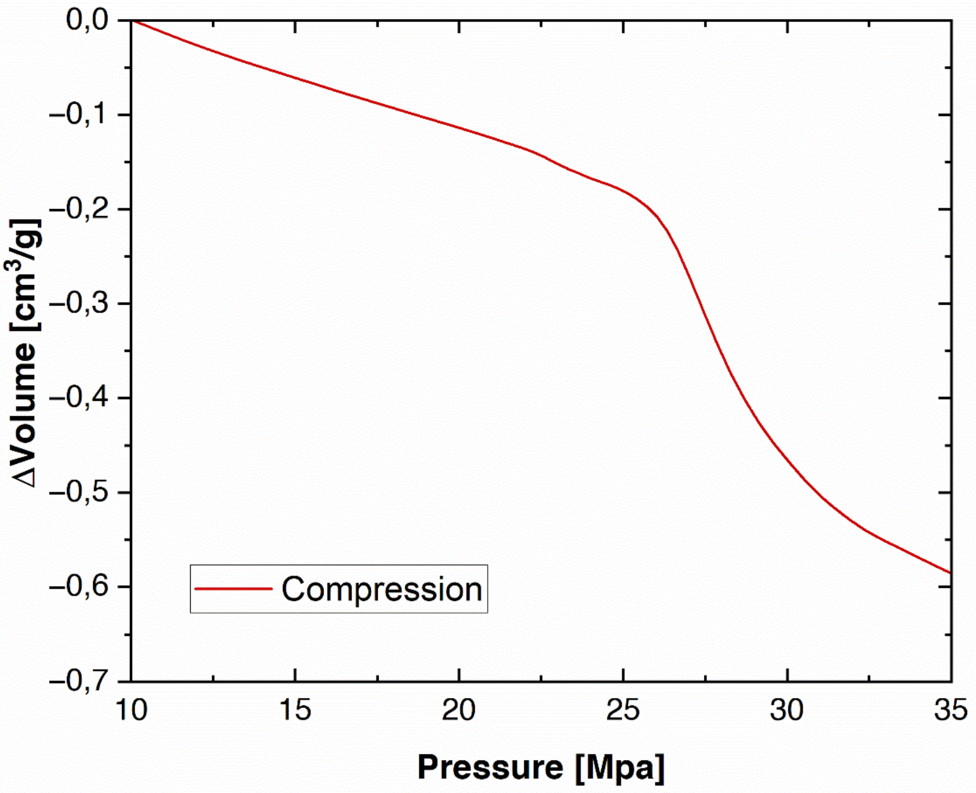

Figure S1. PV-isotherm of ZIF-8 + water system for compression range.

Synchrotron experiments

In-operando pressure synchrotron powder diffraction studies were carried out at beamline 17BM of the Advanced Photon Source (APS, Argonne, USA) [17BM]. Si(311) monochromator was tuned to an incident energy of 27 keV. The resulting wavelength (λ = 0.451810(1) Å) and the detector distance (600 mm) were calibrated using NIST SRM660a LaB6. Diffraction images were collected using Varex 3434CT 2D detector and processed by GSAS-II [GSASII] package.

Powdered sample of ZIF-8 (Sigma Aldrich Basolite 1000, LOT S45328-308) was loaded into a sapphire capillary with a K-type thermocouple inserted into the powder but outside the region of the beam. The sample was held in place with pieces of kapton tube and quartz wool. The temperature was maintained using Oxford Cryosystems Cryostream nitrogen blower on the basis of the readout from the thermocouple.

Pressure in the system was dynamically stabilized by an ISCO syringe pump which was filled with Water ASTM Type II (VWR Chemicals BDH, BDH1168-4LP, LOT 20H1156905).

The sample was activated in-situ by flowing He gas at 100oC for 20 minutes. Then the sample was cooled down to ambient temperature and flooded with water. Several XRDs collected before the activation, during activation, post activation at RT and after flooding with water at the RT did not reveal any signs of decomposition.

The measurements were carried out by collecting a single dark frame and 4 data frames (0, 1, 2, 3) at each pressure. Each frame lasted 30 seconds and the first 3 frames (0, 1, 2) were collected when system was equilibrating to isothermal conditions. Only the frames number 3 were used for the analysis.

The data were collected with 1 MPa step before and after the intrusion, while 0.5 MPa step was used in the intrusion/extrusion region.

The effective pressurization and depressurization rates were 0.39 MPa/min pre- and after the intrusion and 0.19 MPa/min during the (in/ex)trusion.

Pattern matching analysis was carried out using Fullprof suite of programs [FP] (May 2021) and the serial refinements were automated using Python scripts.

The LeBail analysis was used to extract lattice parameters and estimate effects of peak broadening due to domain size effects and strain induced by the (in/ex)trusion.

The coherent domain size and strain were estimated using models built-in into the Fullprof suite. The instrumental resolution file was obtained from the LaB6 standard used for calibration. The diffraction line profile was parametrized using the Thompson-Cox-Hastings profile [TCH]. Although the original physical background of the TCH profile is not fully reflected in the 2D detectors [SNBL], it still provides very good parameterization of the whole diffraction profile. The size and size broadening in imposed on top of the machine resolution, so in the first order approximation it does not depend on a particular baseline shape. The anisotropic strain broadening was parametrized in the quartic form [AnisStephens] and the anisotropic Lorentzian size broadening was modeled using spherical harmonic approach proposed by Jarvinen [Jarvi]. The models appropriate for the Laue class m-3m (Size=17, Strain=13) were used and the effective size and strain were calculated by the Fullprof.

Atomistic simulations

RMD simulations

A comprehensive observation of the wetting and filling process of the ZIF-8 slab is precluded by the typical timescale of ordinary molecular dynamics. An experimental intrusion process can be observed over fractions of a second in some materials while typical long molecular dynamics simulations are restricted to hundreds of nanoseconds at maximum. Thus, to accelerate the sampling of slab filling, RMD (Restrained Molecular Dynamics) simulations were performed, and the free energy associated to the process computed.[1,2]

In the present work, we applied RMD techniques for three different scenarios:

1. The filling of the entire ZIF-8 slab by water molecules in order to evaluate the wetting mechanism of the 28 cages comprising the slab.
2. The wetting of a single cage: in this case the free energy of process is also presented.
3. The filling (and free energy) of the same cage studied in scenario 2 but restricting water entry to one 6MR aperture.

In statistical mechanics, a thermodynamic potential is connected to the logarithm of a suitable probability density function in the chosen ensemble. In the present cases, the relevant probability density function is expressed as $M(N^*_{\mathrm{H_2O}})$, the probability density that a certain number of H_2O molecules are inside the slab (scenario 1) or inside a given cage (scenario 2-3). Introducing the ensemble distribution $m(\boldsymbol{r})$, namely the probability density to find the atoms of the system in position corresponding to the $3N$ dimensional vector $\boldsymbol{r}$, it is possible to express $M\left(N^*_{\mathrm{H_2O}}\right)$ as:

$$M\left(N^*_{\mathrm{H_2O}}\right) = \int d\boldsymbol{r}\, m(\boldsymbol{r})\, \delta\left(\hat{N}_{H_2O}(\boldsymbol{r}) - N^*_{H_2O}\right) \qquad \text{[S1]}$$

Where $\delta(\ \cdot\ )$ is the Dirac delta function and $\hat{N}_{H_2O}(\boldsymbol{r})$ is the number of water molecules inside the ZIF-8 slab (or the cage) in the atomistic configuration $\boldsymbol{r}$ and $N^*_{H_2O}$ is the target value. Under this condition, it is possible to associate the Landau free energy, $G(N^*_{H_2O})$, with the probability density as following:

$$G(N^*_{H_2O}) = -k_B T \log M(N^*_{\mathrm{H_2O}}) \qquad [S2]$$

where $k_B T$ is the thermal energy at the experimental temperature T (k_B is the Boltzmann constant).

To count the number of H_2O molecules we need to define the spatial region of ZIF-8 slab and the volume of space enclosed inside the cage. In the case of slab, it turns out to be simple because is directly connected to the size of slab. For the volume of the cage, it is necessary to prevent the volume of the sphere from encroaching on that of adjacent cages. So, we start by defining the sphere located at the centre of the cage with radius equal to the average distance between the centre and the Zn atoms comprising the cage. This sphere partially intersects the volume of neighbouring cages. Indeed, a sphere sufficiently large to enclose the truncated octahedral cage has a radius equal to $\sqrt{15}/5$ of the average distance between the cage centre and its Zn atoms. This is the final cage volume we define to check the number of water molecules in the relevant cavity.

In principle, one can compute $M(N^*_{\mathrm{H_2O}})$ by running a long simulation and calculating the histogram of $N^*_{\mathrm{H_2O}}$ along it: the histogram is a decent approximation of $M(N^*_{\mathrm{H_2O}})$, from which one can compute the free energy using Eq. [S1]. However, this approach results as a very inefficient computational procedure, because, with large free energy barriers to intrusion (much higher than the thermal energy $k_B T$), within the typical MD simulation time the system can sample only configurations similar to the initial one.

This problem can be overcome using RMD.[3,4] Inside the RMD framework, a controlled bias is introduced to force the system to explore configurations corresponding to a specific number of molecules intruded in our slab or single cell. Starting from the Landau free energy (Eq. S1) and, for the sake of simplicity, assuming that the ensemble is canonical, we have:

$$m(\boldsymbol{r}) = \left. exp\left[-{}^{V(\boldsymbol{r})}\!/_{k_B T}\right] \middle/ \int d\boldsymbol{r}\, exp\left[-{}^{V(\boldsymbol{r})}\!/_{k_B T}\right] \right. \qquad [S3]$$

where $V(\boldsymbol{r})$ is the (physical) interacting potential: the *force field*. Within the canonical ensemble the probability density function of Eq. [S1] to have $N^*_{\mathrm{H_2O}}$ water molecules within a prescribed volume, the entire slab of a single cage, reads:

$$M(N^*_{\mathrm{H_2O}}) = \frac{\int dr\, exp\left[-{}^{V(\boldsymbol{r})}\!/_{k_B T}\right]\delta\left(\hat{N}_{H_2O}(\boldsymbol{r}) - N^*_{H_2O}\right)}{\int dr\, exp\left[-{}^{V(\boldsymbol{r})}\!/_{k_B T}\right]} \qquad [S4]$$

The derivative of the free energy is:

$$\frac{dG(N^*_{\mathrm{H_2O}})}{dN^*_{\mathrm{H_2O}}} = -\,k_B T \frac{\int dr\, exp\left[-{}^{V(\boldsymbol{r})}\!/_{k_B T}\right] {}^{d\left[\delta\left(\hat{N}_{H_2O}(\boldsymbol{r}) - N^*_{H_2O}\right)\right]}\!/_{dN^*_{\mathrm{H_2O}}}}{\int dr\, exp\left[-{}^{V(\boldsymbol{r})}\!/_{k_B T}\right]\delta\left(\hat{N}_{H_2O}(\boldsymbol{r}) - N^*_{H_2O}\right)} \qquad [S5]$$

$G(N^*_{\mathrm{H_2O}})$ can be computed by numerical integration of ${}^{dG(N^*_{\mathrm{H_2O}})}\!/_{dN^*_{\mathrm{H_2O}}}$. The advantage being that the derivative of free energy can be more easily estimated via atomistic simulations. To achieve this objective, in Eq. [S5] one replaces the Dirac delta functions with a smooth Gaussian approximation:

$$\delta\left(\hat{N}_{H_2O}(\boldsymbol{r}) - N^*_{H_2O}\right) \sim g_\lambda\left(\hat{N}_{H_2O}(\boldsymbol{r}) - N^*_{H_2O}\right) = \sqrt{2\pi k_B T/\lambda}\; exp\left[-\frac{\lambda}{2}{}^{\left(\hat{N}_{H_2O}(\boldsymbol{r}) - N^*_{H_2O}\right)^2}\!/_{k_B T}\right] \qquad [S6]$$

Here, $k_B T/\lambda$ is the variance of the Gaussian function, the parameter determining its width, and thus the accuracy of the approximation of the corresponding Dirac delta function. Within the Gaussian approximation the derivative of the free energy, Eq [S5], reads:

$$\frac{dG(N^*_{\mathrm{H_2O}})}{dN^*_{\mathrm{H_2O}}} \sim \frac{\int dr\, \lambda\left(\hat{N}_{H_2O}(\boldsymbol{r}) - N^*_{H_2O}\right) exp\left\{-\left[V(\boldsymbol{r}) + \frac{\lambda}{2}\left(\hat{N}_{H_2O}(\boldsymbol{r}) - N^*_{H_2O}\right)^2\right] \Big/ k_B T\right\}}{\int dr\, exp\left\{-\left[V(\boldsymbol{r}) + \frac{\lambda}{2}\left(\hat{N}_{H_2O}(\boldsymbol{r}) - N^*_{H_2O}\right)^2\right] \Big/ k_B T\right\}} \qquad [S7]$$

Thus, the derivative of the free energy can be computed as the expectation value of $\lambda\left(\hat{N}_{H_2O}(\boldsymbol{r}) - N^*_{H_2O}\right)$ over the canonical ensemble of a system driven by the so-called augmented potential $V(\boldsymbol{r}) + \frac{\lambda}{2}\left(\hat{N}_{H_2O}(\boldsymbol{r}) - N^*_{H_2O}\right)^2$.

In practice, it is possible to compute $dG(N^*_{\mathrm{H_2O}}) \big/ dN^*_{\mathrm{H_2O}}$ at each desired value of $N^*_{\mathrm{H_2O}}$ by averaging the observable $\lambda\left(\hat{N}_{H_2O}(\boldsymbol{r}) - N^*_{H_2O}\right)$ along MD trajectory driven by the potential $V(\boldsymbol{r}) + \frac{\lambda}{2}\left(\hat{N}_{H_2O}(\boldsymbol{r}) - N^*_{H_2O}\right)^2$. Finally, the $dG(N^*_{\mathrm{H_2O}}) \big/ dN^*_{\mathrm{H_2O}}$ so obtained is numerically integrated by the trapezoid rule. The free energy landscape of filling one single cage molecule by molecule is reported in main text Figure 2c (blue curve).

The RMD framework can also be used to prevent water molecules from passing through 6MR and forming hydrogen bonds between two different cages. In this case, we define a spherical volume at the centre of 6MR apertures of radius 1.5 Å and we impose that the target value $N^*_{\mathrm{H_2O}}$ must be equal to zero inside the spherical volume. By imposing a similar "repulsive" bias in the middle of seven of the 6MR apertures belonging to the same cage (each cage has 8 different 6MR apertures) we can compute the free energy profile of the filling of the cage when water molecules can

enter through only one 6MR aperture and the *hydrogen bond bridge* formation through all the other 6MR is disrupted (see main text figure 2c red curve).

Since the free energy is the result of numerical integration of the so-called mean force, which in turn is the expectation of an observable over the biased ensemble average of a suitable observable, the free energy is affected by statistical error. The statistical error on a derived observable $O = O(s)$, with s the directly measured variable. Here the free energy and mean force statistical error, respectively, is usually obtained by error propagation:

$$\delta O^2 = \left(\frac{dO(s)}{ds}\delta s\right)^2 \qquad [S8]$$

Here, δs^2 and δO^2 are the variance of s and the estimated of O, respectively.

In free energy calculations thermodynamic integration or analogous techniques, e.g., RMD, where the free energy is obtained by numerical integration of $dG(N^*_{\mathrm{H_2O}}) / dN^*_{\mathrm{H_2O}}$, error propagation leads to a severe overestimation of $dG(N^*_{\mathrm{H_2O}})$:

$$\delta G(N_j)^2 = \sum_{i=1,j} \frac{G'(N_i)^2 + G'(N_{i-1})^2}{2}(N_i - N_{i-1})^2 \qquad [S9]$$

where $G'(\cdot)$ is a compact notation of $dG(N^*_{\mathrm{H_2O}}) / dN^*_{\mathrm{H_2O}}$, N_i represents the number of water molecules in the ZIF-8 slab or cage.

Here, like in previous works,[5,6] we implemented a different approach. We divide the configurations used to estimate $dG(N^*_{\mathrm{H_2O}}) / dN^*_{\mathrm{H_2O}}$ in M smaller sets wherefrom we calculate the corresponding estimates of the mean force $\left\{\left(dG(N^*_{H_2O}) / dN^*_{H_2O}\right)_i\right\}_{i=1,M}$.

For each series of free energy gradients, across all values of $N^*_{H_2O}$, one obtains a set

of free energy curves $\{(G(N^*_{\mathrm{H_2O}}))_i\}_{i=1,M}$, via numerical integration which can be used to directly compute the variance $\delta G(N_j)^2$ at each number N_j of water molecules in the cage:

$$\delta G(N_j)^2 = \frac{1}{M-1}\sum_{i=1,M}\left[G((N_j))_i - G(N)_j\right]^2 \qquad [S10]$$

Here, $G(N_j)$ is obtained from the numerical integration of $dG(N^*_{\mathrm{H_2O}}) \big/ dN^*_{\mathrm{H_2O}}$ determined by the complete set of simulation data. Correlation effects can be properly considered by using standard techniques such as the *block average* or the *Jack-knife* methods.[7]

The resulting error values for each point of the two free energy profiles are visualised in figure 2c as the two coloured shades in the background.

Computational details

An oriented ZIF-8 slab along the (100) direction is implemented in our computational system, consisting of a 7x2x2 supercell: 7 layers, each made up of 4 cages, creating a 28-cage sample (visualised in figure 2a of the main text). The slab is immersed in bulk water consisting of 4800 water molecules. The simulations were carried out using an NPT *ensemble*, in which the total number of particles, the applied pressure, and the temperature were keep constant. A 25 MPa pressure were applied through two pistons parallel to the slab, in line with the method proposed by Marchio *et al.*[8] Force-field parameters characterising the interaction potentials of ZIF-8 followed those recently published in Zheng *et al*.[9] The TIP4P/2005 model was used to describe the interaction of the water molecules. All the simulation were performed using LAMMPS package.

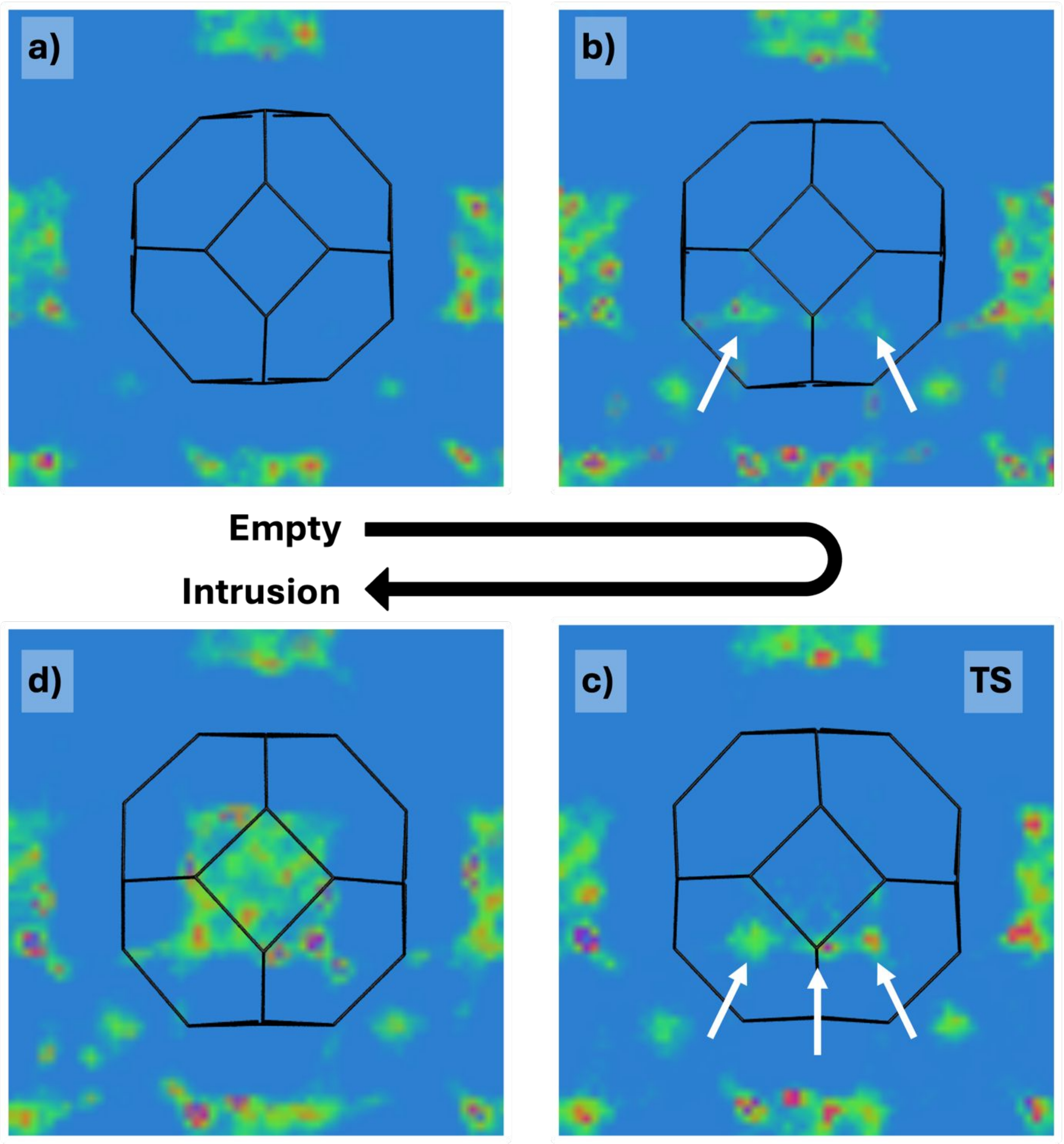

Figure S2 – Density maps of water inside the cage filled using RMD simulations. All profiles are shown at the same height of the cage in order to catch the present of water molecules at the middle of 6MR apertures. a) In the empty cage, no signal of the presence of water molecules is reported. b-c) are the density maps before (b, 5 water molecules in the cage) and when (c, 8 water molecules in the cage) the system reaches the transition state. This figure highlights, beyond the information provided by a single snapshot of the atomistic configuration of Fig. 2d-g, that the transition state of the wetting of single cages corresponds to the formation of hydrogen bonds between files of water molecule entering from wet adjacent cages (put in

evidence by the white arrows in panels b and c) The panel d) reports the water map density when the system reaches the minimum in the energy and the cage is completely filled.

Stabilization effect of hydrogen bonding across 6MR windows

Stabilization of completely filled cages and filling cages at the transition state is obtained from the comparison of the free energy curves of Fig. 2c.

First, we computed the difference of free energies at the barrier of Figure 2c, $\Delta\Delta F$, divided it by the number of surrounding wet cages minus one, n -1, i.e., the number of 6MR apertures which allow water to intrude the considered one: $\Delta\Delta F^{\dagger}/(n-1) \sim 1.9$ kBT is the reduction of the wetting barrier per adjacent wet cage. An analogous calculation can be done to obtain the stabilization effect on the fully wet cage due to the presence of adjacent wet cages, $\Delta\Delta F/(n-1) \sim 6.5$ kBT. These values are used in the coarse-grained stochastic model of intrusion.

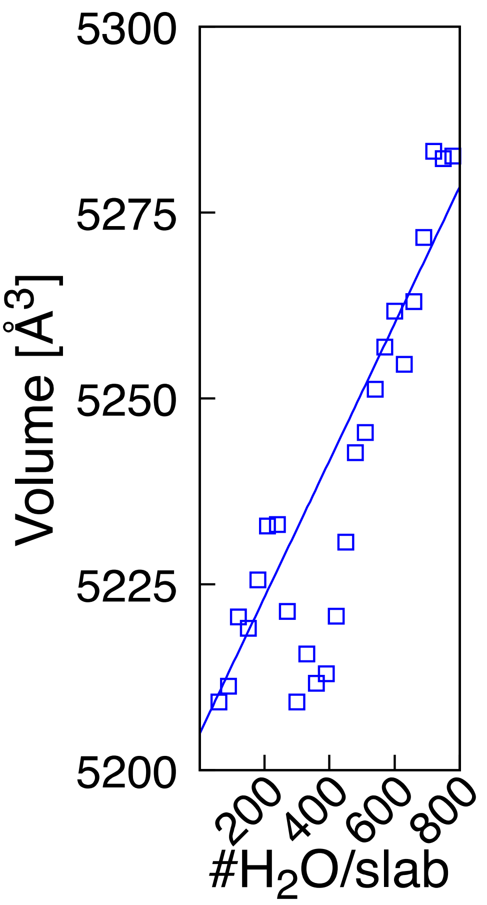

Figure S3. Average volume of a ZIF-8 cage along intrusion, measured by the number of water molecules in the ZIF-8 computational slab. We remark that this value is computed as the time average along restrained molecular dynamics of the volume of the slab at each level of filling divided by the number of cages composing the slab. One notices that the average cell volume continuously grows along intrusion, which is the result of the increasing number of wet cages.

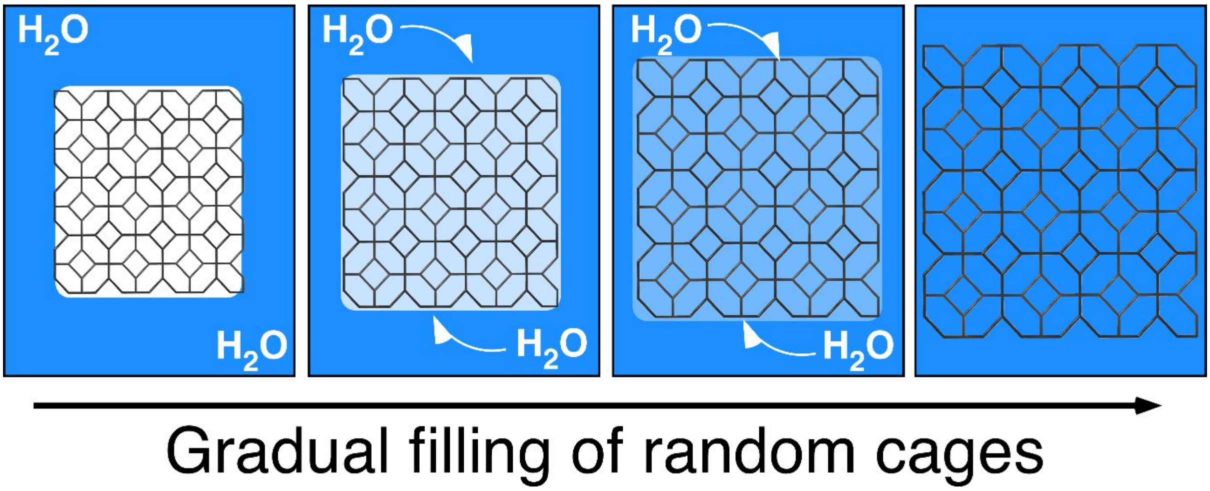

Figure S4. Schematic model of the mechanism of water condensation in random ZIF-8 cavities. This scenario is opposite to our observations, which is shown schematically in Fig. 1 in the manuscript.

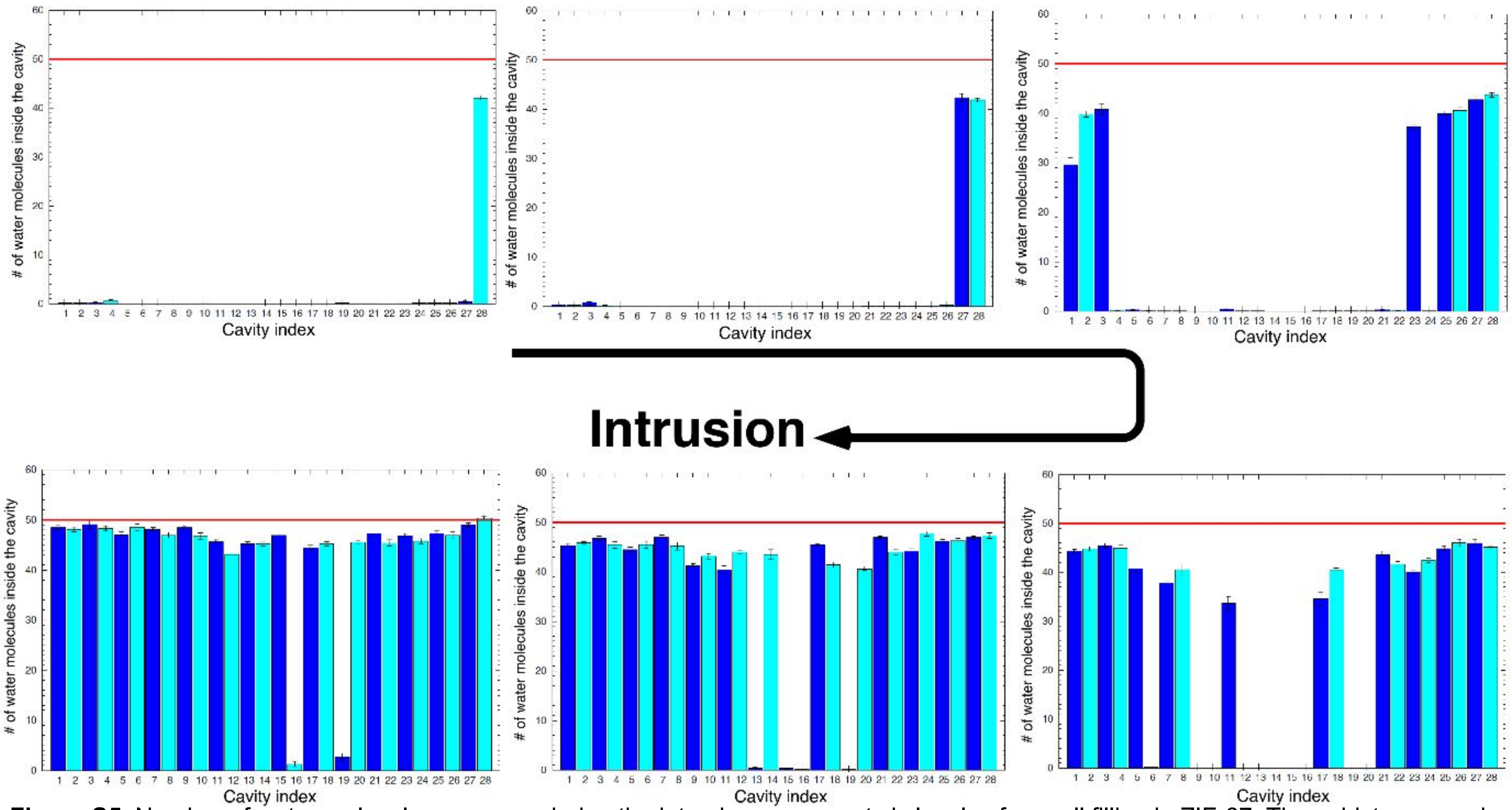

Figure S5. Number of water molecules per cage during the intrusion process at six levels of overall filling in ZIF-67. These histograms show that intrusion starts from cages in contact with bulk water and proceeds toward the interior through a cascade, confirming cage-by-cage filling mechanism in ZIF-67, isomorph material to ZIF-8.

Stochastic model

To simulate the intrusion process in a ZIF-8 crystallite we designed a stochastic model that represents the material as a lattice with the correct number of neighbors, namely being connected through the corners of the lattice and not to the first neighbors.

In this model each site of the lattice is either filled or empty, corresponding to each ZIF-8 cage being intruded or not. We then consider that the filling/emptying rate is follows an Arrhenius-like law:

$$t_f = t_f^0 e^{\frac{\Omega_f}{k_B T}} \quad \text{[S11]}$$

$$t_e = t_e^0 e^{\frac{\Omega_e}{k_B T}} \quad \text{[S12]}$$

Where $t_{f/e}^0$is the characteristic time of filling/emptying, $\Omega_{f/e}$ is the filling/emptying barrier, and k_BT is the thermal energy at operative conditions, with k_B the Boltzmann constant and T the temperature. To minimize the number of parameters we consider t_f^0 and t_e^0 to be the same and independent of pressure, which may not always be true but does not give qualitatively different results.

The results obtained in molecular dynamics gave us indication that the filling/emptying barrier depends on the number of surrounding filled cages and so we model them has:

$$\Omega_x^n(P) = \Omega_x^0(P_0) + \Delta\Omega_x(P - P_0) \pm n\, A_{6MR}\, \gamma \quad \text{[S13]}$$

Here, $\Omega_x^n(P)$ is the barrier of either the filling or emptying process at the operative pressure P, when the cavity has n filled neighbours. $\Omega_x^0(P_0)$ is the corresponding barrier at the reference pressure P_0 and when there are no filled neighbours. $n\, A_{6MR}\, \gamma$ is the energy gain or penalty along filling and emptying, respectively, where A_{6MR} is the area of the 6MR apertures and γ is the surface tension of water when you have a number, n, of already filled cavities around the one undergoing the process. Previous work[2]

shows that $\Delta\Omega_f(P-P_0)$=$(P-P_0)*v_g$ with v_g the volume of the critical gas bubble during the filling of the cavity. For the complementary emptying process, a similar term is found $\Delta\Omega_e(P-P_0)=(P-P_0)*(v-v_g)$,where v is the total volume of the cavity. Following the same work, we find a relation between Ω^0_{empty} and Ω^0_{fill}:

$$\Omega^0_e = \Omega^0_f + \gamma\cos\theta \mathrm{A} + (2nA_{6MR} - 8A_{6MR})\gamma + P\,v \quad [S14]$$

Where $\cos\theta$ is the (effective) contact angle of ZIF-8 cavities, and A is the contact area. Our model has five parameters: $\Omega^0_{f,}$ v_g, A_{6MR}, $\cos\theta A$, and v. Here, we chose reasonable values for these parameters, namely v_g = 0.1 k_BT/MPa, v = 0.4 k_BT/MPa, A_{6MR}* γ = 2.5 k_BT, cos θ * A_{sv}*γ = -8 k_BT, Ω^0_f = 25 k_BT. Some pressures or number of neighbours could lead to negative barriers, in these cases we set them to zero and the system only has one possible state, either filled or empty.

With this model for the filling and emptying rates we change the filling state of each cell according to a probability that depends on the corresponding transition rate:

$$p_f = 1 - e^{-dt/k_f} \quad [S15]$$

$$p_e = 1 - e^{-dt/k_e} \quad [S16]$$

Eq. [S15] and [S16] are the result of the integration of the first order kinetic process. For dt, the *timestep* we used in the numerical simulation of our stochastic process, is set to 10000 times the value of t^0_f and t^0_e, which in other works[10,11] regarding wetting of hydrophobic nanopores was c.a. 10^{-10}-10^{-12}s. In our simulations, we modelled the experimental scanning of pressure from 0 MPa to 50 MPa in a continuous, linear manner for 50000 timesteps, equivalent to a cycle of 5 ms. We initialize the 3D grid with all the cells empty and we consider that the surface cells are in contact with bulk water, meaning they have wet neighbours.

We refrain the reader to strictly apply to stochastic model to interpret experimental results: crystalline grains and morphology, defects, pressure scanning rate and other aspects are different between the stochastic model and experiments. Hence, we draw the attention of the reader on the general and qualitative aspects of the stochastic model results, rather than in the quantitative details.

References

[1] J. B. Abrams, M. E. Tuckerman, *Journal of Physical Chemistry B* **2008**, *112*, 15742–15757.

[2] L. Maragliano, E. Vanden-Eijnden, *Chem Phys Lett* **2006**, *426*, 168–175.

[3] S. Bonella, S. Meloni, G. Ciccotti, *The European Physical Journal B 2012 85:3* **2012**, *85*, 1–19.

[4] S. Meloni, G. Ciccotti, *The European Physical Journal Special Topics 2015 224:12* **2015**, *224*, 2389–2407.

[5] M. Tortora, P. Zajdel, A. R. Lowe, M. Chorążewski, J. B. Leão, G. v. Jensen, M. Bleuel, A. Giacomello, C. M. Casciola, S. Meloni, Y. Grosu, *Nano Lett* **2021**, *21*, 2848–2853.

[6] W. Janke, *Lecture Notes* **2002**, *10*, 423–445.

[7] M. Amabili, S. Meloni, A. Giacomello, C. M. Casciola, *Journal of Physical Chemistry B* **2017**, *122*, 200–212.

[8] S. Marchio, S. Meloni, A. Giacomello, C. M. Casciola, *Nanoscale* **2019**, *11*, 21458–21470.

[9] B. Zheng, M. Sant, P. Demontis, G. B. Suffritti, *Journal of Physical Chemistry C* **2012**, *116*, 933–938.

[10] L. Guillemot, T. Biben, A. Galarneau, G. Vigier, É. Charlaix, *Proc Natl Acad Sci U S A* **2012**, *109*, 19557–19562.

[11] A. Tinti, A. Giacomello, Y. Grosu, C. M. Casciola, *Proc Natl Acad Sci U S A* **2017**, *114*, E10266–E10273.
